# The Natively Disordered Loop of Bcl-2 Undergoes Phosphorylation-Dependent Conformational Change and Interacts with Pin1

**DOI:** 10.1371/journal.pone.0052047

**Published:** 2012-12-18

**Authors:** CongBao Kang, Nagakumar Bharatham, Joel Chia, Yuguang Mu, Kwanghee Baek, Ho Sup Yoon

**Affiliations:** 1 Division of Structural and Computational Biology, School of Biological Sciences, Nanyang Technological University, Singapore, Singapore; 2 Experimental Therapeutics Centre, Agency for Science, Technology and Research, Singapore, Singapore; 3 Department of Genetic Engineering, College of Life Sciences, Kyung Hee University, Yongin-si, Gyeonggi-do, Republic of Korea; University of South Florida College of Medicine, United States of America

## Abstract

Bcl-2 plays a central role in the regulation of apoptosis. Structural studies of Bcl-2 revealed the presence of a flexible and natively disordered loop that bridges the Bcl-2 homology motifs, BH3 and BH4. This loop is phosphorylated on multiple sites in response to a variety of external stimuli, including the microtubule-targeting drugs, paclitaxel and colchicine. Currently, the underlying molecular mechanism of Bcl-2 phosphorylation and its biological significance remain elusive. In this study, we investigated the molecular characteristics of this anti-apoptotic protein. To this end, we generated synthetic peptides derived from the Bcl-2 loop, and multiple Bcl-2 loop truncation mutants that include the phosphorylation sites. Our results demonstrate that S87 in the flexible loop of Bcl-2 is the primary phosphorylation site for JNK and ERK2, suggesting some sequence or structural specificity for the phosphorylation by these kinases. Our NMR studies and molecular dynamics simulation studies support indicate that phosphorylation of S87 induces a conformational change in the peptide. Finally, we show that the phosphorylated peptides of the Bcl-2 loop can bind Pin1, further substantiating the phosphorylation-mediated conformation change of Bcl-2.

## Introduction

Apoptosis is an essential physiological process for the development and homeostasis of multi-cellular organisms [1]. It is a well-orchestrated and highly controlled cellular process, regulated by pro- and anti-apoptotic proteins [2]. The Bcl-2 family of proteins is central regulators in the mitochondrial-mediated apoptotic pathways [3], [4]. Three-dimensional structural studies of the anti-apoptotic proteins, Bcl-2 and Bcl-X_L_, have revealed the presence of a highly flexible and intrinsically disordered loop between Bcl-2 homology motifs, BH3 and BH4 [5], [6]. Bcl-2 is phosphorylated at multiple sites in the flexible loop (T56, S70, T74, S87) in response to various external stimuli [7], [8], [9]. Kinases, such as c-jun N-terminal kinase (JNK) and extracellular signal-regulated kinase 2 (ERK2), are involved in Bcl-2 phosphorylation [7], [10], [11], [12], and the phosphorylation appears to regulate the activity of Bcl-2. On the other hand, phosphatases, such as protein phosphatase (PP)1, PP2A and PP2B [13] can also interact with Bcl-2 and modulate its activity [14]. All of the known phosphorylation sites in the flexible loop of Bcl-2 contain Ser/Thr-Pro motifs [7], which are phosphorylated by Pro-directed protein kinases and exist in *cis* and *trans* isomers, the conversion of which can be catalyzed by peptidylprolyl *cis-trans* isomerases (PPIase) [15]. Peptidylprolyl *cis-trans* isomerization of some proteins serves as a molecular switch and influences protein function [16].

Human Pin1 is a member of the parvulin family, and its homologues catalyze the isomerization of phosphorylated Ser-Pro or Thr-Pro peptide bonds [17], which provides important regulatory steps in various cellular processes, including the cell cycle [17]. The N-terminal WW domain of Pin1 is responsible for protein-protein interactions. Recent studies suggest that Pin1 interacts with the phosphorylated Bcl-2, and the location of phosphorylated Bcl-2 might be controlled by Pin1 [18]. Taken together, Bcl-2 phosphorylation appears to be coordinated in a complex, dynamic network, with the participation of multiple targets. In this work, to further define and better understand the biological significance and the regulation of Bcl-2 through the phosphorylation of its disordered loop, we have studied the specificity of relevant kinases and phosphatases that have been shown to be associated with the phosphorylation and dephosphorylation of Bcl-2. The interaction between Pin1 and phosphorylated peptide from the loop region of Bcl-2 was also studied. Our NMR and computational studies provide evidence to suggest that phosphorylation in the disordered loop of Bcl-2 drives a conformational change that consequently allows its molecular interaction with Pin1.

## Materials and Methods

### Materials

An antibody against Bcl-2 was purchased from Santa Cruz Biotechnology (Santa Cruz, CA, USA). RNeasy Mini Kit was from Qiagen (Hilden, Germany). Immun-Star ™ chemiluminescent protein detection system and protein molecular weight marker were from Bio-Rad Laboratories (Hercules, CA, USA). PP2A was from Upstate (Lake Placid, NY, USA). γ-^32^P-ATP (3000 Ci/mmol) and HiPrep 16/60 Sephacryl S-200 were from Amersham Biosciences (Buckinghamshire, UK). PMSF, RT-PCR kit and other restriction enzymes were from Roche (Indianapolis, IN, USA). EGTA and other chemicals were purchased from Sigma-Aldrich (St. Louis, Mo, USA). Carbenicillin was from Invitrogen (Carlsbad, CA, USA). All peptides and phosphorylated peptides were synthesized from GL Biochem Ltd (Shanghai, China) and confirmed by Mass analysis. PP1 was from New England Biolabs (Ipswich, MA, USA). PP2B (Calcineurin) and the phosphate assay dye were purchased from BIOMOL (Plymouth Meeting, PA, USA).

### Plasmid construction and protein purification

The cDNA of human Bcl-2 was amplified by reverse transcription-polymerase chain reaction (RT-PCR) using RNA isolated from MCF7 breast cancer cells. cDNA coding for Bcl-2 and Pin1 were digested with *Nde*I and *Xho*I, and inserted into pET16 vector (Novagen, Madison, WI, USA) to give pET-Bcl-2 and pET-Pin1, respectively. The proteins were expressed with a decahistidine (10His) tag at the N-terminus and purified as described previously [19]. Bcl-2Δ(V35–V89):6A, which is the flexible loop-deletion mutant of Bcl-2 was obtained as previously described [19], The plasmids coding for JNK and ERK were a kind gift from Prof. Melanie H. Cobb, and the purification of JNK and ERK2 was performed as described [20]. The plasmid coding for PP2B was a kind gift from Prof. Jun O. Liu, and PP2B was purified as previously described [13].

### Western blot analysis

Proteins were loaded on 12.5% gels and separated by SDS-PAGE before transfer to PVDF membranes. Membranes were first blocked with 1% skim milk in Tris-buffered saline (TBS) buffer (20 mM Tris-HCl, pH 7.5, 500 mM NaCl) and then incubated with anti-Bcl2 in TBS buffer containing 0.2% skim milk for 2 h at 37°C. The immunoreactivity was detected using immun-Star™ chemiluminescent protein detection system (Bio-Rad Laboratories, Hercules, CA, USA).

### Phosphorylation of Bcl-2

For the phosphorylation of Bcl-2 by JNK, the purified Bcl-2 (6 µM) was incubated with 0.5 µM JNK for 1 h at 30°C in a buffer containing 10 mM Tris-HCl pH 7.5, 25 mM MgCl_2_, 1 mM EGTA, 1 mM ATP, 1 µCi γ-^32^P-ATP, and 250 µM PMSF. For the phosphorylation of Bcl-2 by ERK2, ERK2 was incubated with purified Bcl-2 in a buffer containing 50 mM Tris-HCl, 10 mM MgCl_2_, 2 mM dithiothreitol (DTT), 1 mM EGTA, and 0.01% Brij 35 detergent, pH 7.5. The samples were separated on 12.5% gels by SDS-PAGE and visualized using autoradiography.

### Phosphorylation of Bcl-2 loop peptides

The loop peptides (200 µM) were mixed with JNK or ERK2 (10 µM) in the reaction buffer as described above in the “Phosphorylation of Bcl-2”. The reaction was performed at 30°C for 1 h. The samples were passed through a Ni^2+^-NTA column to remove kinases fused with the histidine-tag, and the flow-through samples were combined and loaded on a C-18 column for HPLC analysis. The samples (20 µl) were injected into the C-18 column with a flow rate of 1 ml/min. The peptide was eluted with a linear gradient of acetonitrile from 0 to 30%, and the peptide peaks were detected at 225 nm. The peptides sequences used in this study are shown in Table 1.

**Table 1:** Peptide Sequences.

Peptide	Sequence
T56	QPGH**T**PHPAA
pT56	QPGHp**T**PHPAA
S70	PVART**S**PLQT
pS70	PVARTp**S**PLQT
T74	PLQ**T**PAAPGA
pT74	PLQp**T**PAAPGA
S87	GPAL**S**PVPPV
pS87	GPALp**S**PVPPV

Phosphorylated sites are indicated in bold and numbered according to their positions in the Bcl-2 sequence. Residues phosphorylated are indicated with label “p”.

### NMR spectroscopy

NMR data were acquired at 300 K on a Bruker Avance 600 equipped with a cryoprobe accessory. The NMR samples contained 0.1 mM ^15^N-labeled Pin 1 in 90% H_2_O/10% D_2_O in 20 mM phosphate buffer, pH 7.0, 20 mM NaCl, 1 mM DTT, 0.01% NaN_3_. Chemical shift perturbations were monitored with 2D ^1^H-^15^N heteronuclear single quantum correlation spectroscopy (HSQC) upon addition of peptides. NMR spectra were processed using Bruker Topspin1.3. For the dissociation constant determination, lyophilized aliquots of peptides were mixed with ^15^N-labeled Pin1 with different molar ratios and 2D ^15^N HSQC was recorded. The dissociation constant, *K_D_* is expressed as [P]*[L]/[PL] and calculated as previously described [17], [21], [22].

### Structure determination of peptides

Peptides were dissolved in 20 mM phosphate buffer at pH 6.5. One-dimensional (1D) and two-dimensional (2D) NMR spectra were acquired at 25°C with a Bruker Avance 400 MHz magnet equipped with a BBO probe. 2D TOCSY and ROESY spectra were acquired with 75 ms and 200 ms mixing time. Spectra were processed using Topspin1.3 and NMRpipe [23] and analyzed using NMRView [24]. NOE intensities were classified as strong, medium and weak, and transferred to distance limits to 1.8–2.5, 2.5–3.5 and 3.5–6.0 Å, respectively. Overlapped cross-peaks were used as qualitative restraints of 1.8–6.0 Å. Chemical shift index analysis using the H^α^ showed no secondary structure present in neither S87 nor pS87. A total of 30 NOEs for S87 and 32 NOEs for pS87 were used for the structural calculations. Structure calculations were carried out using XPLOR-NIH [25] with simulated annealing, which was applied using 6000 steps at 1000 K and 100,000 cooling steps to 300 K followed by 50 cycles of powell energy minimization. A total of 50 structures were generated, and the 10 with the lowest energy were selected and visualized in Pymol (www.pymol.org).

### Model of Pin1 WW domain with pS87 Bcl-2 peptide

Several molecular modeling techniques were utilized to develop the initial model of the Pin1 WW domain with the pS87 Bcl-2 peptide. Molecular structures of the Pin1 WW domain with phosphopeptides solved by either X-ray crystallography or NMR methodologies were thoroughly analyzed. Among them, three Pin1 WW domain structures with phosphopeptides, such as the C-terminal domain (CTD) of RNA polymerase II, a phosphothreonine peptide from CDC25, and a phosphothreonine peptide from human tau (PDB ID : 1F8A, 1I8G, and 1I8H respectively) were considered for analyses to select suitable three-dimensional (3D) structures to build the initial model. Based on these analyses, the 1F8A was selected as a suitable complex to build the pS87 Bcl-2 peptide model, as the phosphopeptide in 1F8A possessed a phosphoserine-proline motif, unlike the others. Residues of the phosphopeptide in the 1F8A complex were replaced with the Bcl-2 pS87 peptide sequence (GPALpSPVPPV) using a protein builder module incorporated in Discovery studio 2.1 software, and the complex (Pin1 with pS87 peptide) was minimized thoroughly using the steepest descent minimization method to remove any unfavorable interactions. This initial model was then considered as the starting structure for MD simulation studies.

### Molecular dynamics (MD) stimulations

All MD simulations were performed with the GROMACS [26] program package and the OPLSAA [27] force field employed in GROMACS. The partial charges for the phosphoserine were taken from previous work [28] as well as from input parameters of independent simulations carried out in Desmond molecular simulation package using OPLSAA force field. In this study, we performed several simulations of un-phosphorylated and phosphorylated S87 Bcl-2 peptide to understand the phosphorylation effect on the peptide conformation. We also carried out MD simulations to understand the interactions and binding pattern of phosphorylated Bcl-2 peptide (pS87) with WW domain (N-terminal domain) of Pin1 at the molecular level. The N- and C-terminal residues of the peptide and protein were capped with acetyl (ACE) and N-methyl (NME), respectively, to keep them neutral at the time of simulation. In each simulation, the solute (peptides/peptide with Pin1) was solvated in a rectangular box of SPC water. To neutralize the system, counter-ions were added and water molecules were removed if they overlapped with the ions. The systems contained 2,663 and 16,422 atoms for peptide alone and for complex (peptide with Pin1) simulations, respectively. The equation of motion was integrated using a leapfrog algorithm with a time step of 2 fs. Covalent bond lengths involving hydrogen atoms were constrained by the SHAKE algorithm, with a relative geometric tolerance of 0.0001. A cut-off of 10 Å was used for the non-bonding van der Waals interactions, and the non-bonded interaction pair-list was updated every 10 fs. Periodic boundary conditions were applied, and the particle mesh Ewald method was used to treat electrostatic interactions. The solute and solvent were separately weakly coupled to external temperature baths at 300 K with a temperature coupling constant of 0.5 ps. The total system was also weakly coupled to an external pressure bath at 1 atm using a coupling constant of 5 ps. All systems were minimized and equilibrated with the same protocol, using the program MDRUN in single precision. The whole system was first minimized for 1000 steps. A 100 ps MD run of the water molecules and counter ions with fixed solute was then performed, followed by a 100 ps MD run without position constrains of the solute. The simulations were then continued for 20 ns for peptide alone systems, and 10 ns for complex systems, where the coordinates were saved at each ps for analysis.

## Results

### Kinases show specificity on the phosphorylation of Bcl-2

Bcl-2 is phosphorylated at multiple sites (T56, S70, T74, and S87), in response to different stimuli [7]. JNK and ERK2 have been repeatedly shown to be associated with the Bcl-2 phosphorylation [29]. However, the molecular characteristics of the Bcl-2 phosphorylation by the kinases are unclear. In our current work, we first studied the specificity of JNK and ERK2 on Bcl-2. To better define the specificity of the relevant kinases, we used synthetic peptides encompassing the multiple phosphorylation sites of Bcl-2 in the flexible loop (Table 1; and herein identified by the residue they cover). The peptides were treated by JNK and ERK2, and the degree of phosphorylation was determined by analyzing the differences in the elution profiles between the peptides and phosphopeptides on a HPLC column. From the HPLC elution profiles, we showed that both JNK and ERK2 were able to phosphorylate the T56 peptide to a similar level (Fig. S1A & S1B). The S70 and T74 peptides were poorly phosphorylated by both JNK and ERK2 (Fig. S1A & S1B), whereas the S87 peptide showed preferred phosphorylation by both ERK2 and JNK. To confirm the results from the peptide studies, we generated the single mutant forms of Bcl-2 protein (Bcl-2/T56A and Bcl-2/S87A) and checked their patterns of phosphorylation using γ-^32^P-ATP, followed by autoradiography. Our results demonstrated that JNK-mediated phosphorylation is abolished in the Bcl-2/T56A mutant, but not ERK2-mediated phosphorylation. On the other hand, phosphorylation by both kinases was affected in the Bcl-2/S87A mutant. These data suggest that the residue S87 in the flexible loop is the main site for the Bcl-2 phosphorylation by JNK and ERK2.

### Phosphorylation in the Bcl-2 protein and loop peptides induces a conformational change

**Figure 1:**
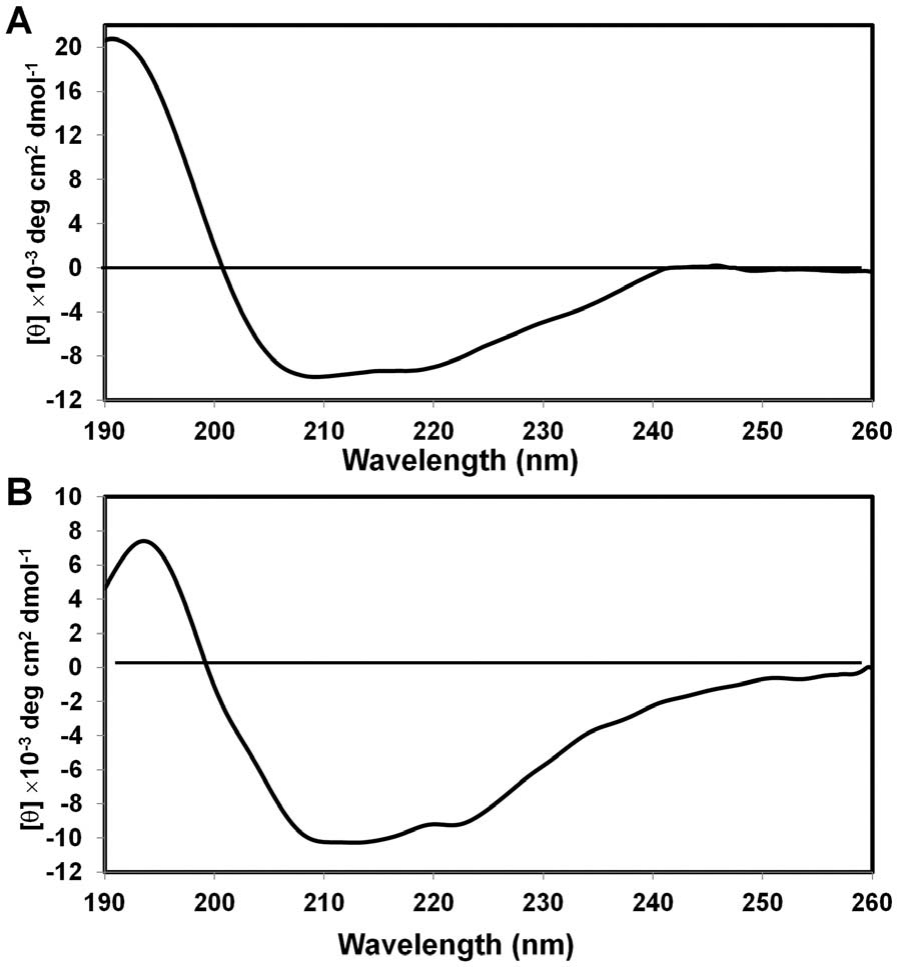
CD analysis of Bcl-2. The purified Bcl-2 was concentrated to 1 mg/ml and exchanged to the phosphate buffer containing 20 Na-PO_4_, pH 7.4, 100 mM NaCl, and subject to CD analysis of Bcl-2 (A) and pBcl-2 (B).

**Figure 2:**
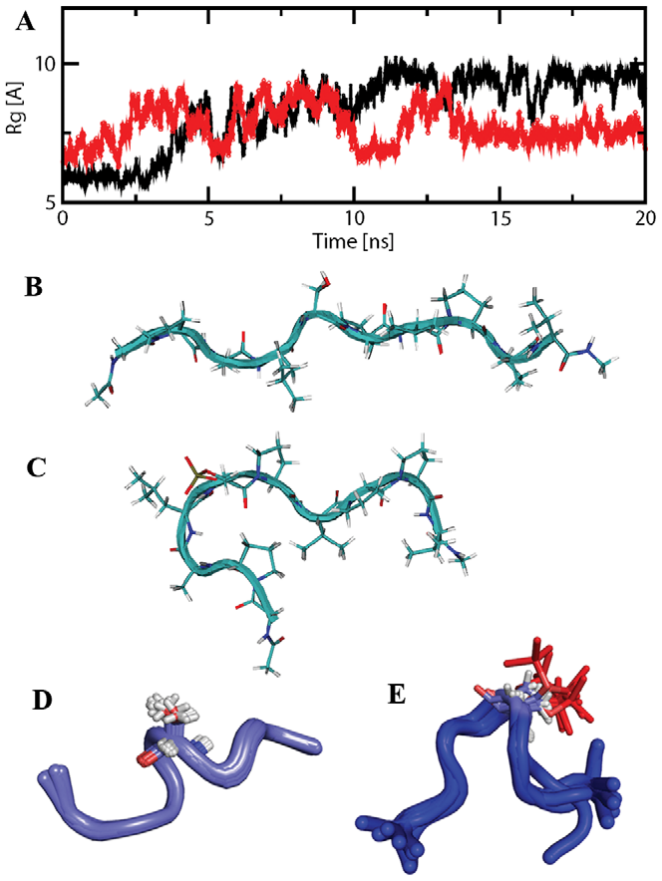
Conformational change of S87 after phosphorylation. (A) Trajectories of radius of gyration (Rg) for peptide S87 in an unphosphorylated (black line) and a phosphorylated (red line) state. (B) Representative structure of the largest-member cluster of unphosphorylated S87 peptide from the final 5 ns simulation trajectory. (C) Representative structure of the largest-member cluster (84%) of phosphorylated S87 peptide from the final 5 ns simulation trajectory. (D) NMR structure ensemble of the 10 lowest energy structures for S87 peptide. (E) NMR structure ensemble of the 10 lowest energy structures for pS87 peptide. Serine and phosphoserine groups are highlighted with sticks.

Our data suggest that the flexible loop of Bcl-2 might have structural dynamics that would regulate the catalytic specificity for kinases and phosphatases. We have tested this possibility by examining any conformational change of the flexible loop of Bcl-2 upon phosphorylation. For this, Bcl-2 and pBcl-2 were purified and their CD profiles compared. Our CD data showed that the random coil content was notably reduced in the pBcl-2 (Fig. 1A & 1B), indicating that the unphosphorylated Bcl-2 undergoes a conformational change upon phosphorylation. This initial observation prompted us to further investigate the conformational change through molecular dynamics simulation. Since S87 showed a preferred site for kinases, in the current work we limited our simulation to the S87 peptide. Three independent simulations starting from different configurations for each phosphorylated and unphosphorylated peptide were performed. The choice of the initial structures was according to the radius of gyration (Rg). First, second and third initial structures have Rg values of 6 Å, 7.5 Å and 9.5 Å, respectively. Three simulations for the unphosphorylated peptide showed a common trend: the Rg of the peptide stabilized around 9.7 Å, suggesting that an extended conformation was the most favored structure for the peptide. As seen in the black line in Fig. 2 (a), the dynamics of the radius of gyration of the unphosphorylated peptide begins with Rg = 6 Å. In the first 3 nanoseconds of simulation, the Rg maintains its value of around 6 Å. Following this, the Rg increases quickly to finally reach equilibrium after 7 ns, and stabilizing at around 9.7 Å after 12 ns. On the other hand, two simulations of the phosphorylated peptide, for which Rg begins with 6 Å and 7.5 Å, showed that Rg stabilized around a value of 7.5 Å. The trajectory of the simulation beginning with Rg = 6 Å is shown in Fig. 2 A in red line. After clustring analysis on the final 5 ns trajectories with RMSD cutoff of 2 Å, the representative structures of the largested-member clusters for normal and phosphorylated peptides are shown in Fig. 2B and C with Rg of 9.6 Å and 6.4 Å, respectively. Fig. 2C shows a snapshot of structure with Rg = 7.5 Å at a simulation time of 20 ns. Clearly, there is a turn around the N-terminus, which makes the overall Rg smaller than the extended structure. The favorable electrostatic interaction between the negatively charged phosphoryl group on the serine 5 and the hydrogen atoms of the backbone amide groups of the same residue and of leucine 4 and alanine 3 (Table 1; number was designated from the N-terminus to C-terminus of the pS87 peptide) may play an important role. This interaction might bring hydrogen atoms of the amide group into proximity with the phosphoryl group of phosphoserine and induce the α_R_ backbone conformation of alanine, leucine and serine. Such helical conformation results in a turn-like overall structure, which is consistent with a recent NMR study [30] and two theoretical molecular dynamics simulation studies [28], [31] of peptide phosphorylation. Moreover, such a turn structure may bring the side chain of proline 2 and side chain of leucine 7 into close contact, which may also provide a stabilization factor. In one simulation of the phosphorylated peptide beginning with Rg = 9.5 Å, such a turn structure did not show up in the 20 ns simulation, which may indicate that the extended-to-turn conformational change is a slow process beyond the 20 ns simulation, and that more extensive simulations will be needed to sample such conformational transitions.

The NMR structures of the S87 and pS87 peptides were also determined based upon the NOE restraints. The proton chemical shift assignment was first achieved from the TOCSY and ROESY spectra (Table S1). Two peptide conformations were observed in the proton 1D spectrum, and we focused on the major form for structure determination (Fig. S4). The chemical shifts, such as amide proton of Ser in pS87, were changed upon phosphorylation (Fig. S4). Chemical shift index analysis using H^α^ showed no secondary structure present in both peptides (Fig. S5). Structure calculation showed that S87 was not a completely extended structure, but no secondary structures, such as an alpha-helix, were observed. This is consistent with the previous result that the loop of Blc-2 is disordered. Upon phosphorylation, pS87 became curved by making the phosphate group facing outward, which would make the SerPro motif easily accessible to some binding partners, such as Pin1 (Fig. 2D, E).

Bcl-2 contains phosphorylation sites mainly restricted to the long flexible loop (residues V35–V89) at T56, T74 and S87 (Fig. S1). Thus, to understand the phosphorylation-mediated conformation of Bcl-2, the effects of phosphorylation on other two regions were also investigated. NMR structures of peptides (T56 and T74) and their cognate phosphorylated peptides (pT56 and pT74) showed similar molecular characteristics as observed with S87 peptide (Fig. S2): after phosphorylation, the phosphorylated peptides also became curved. Taken together, the results suggest that the notable CD change observed in Bcl-2 before and after phosphorylation (Fig. 1) may be due to the fact that the flexible loop harboring major phosphorylation sites undergoes the phosphorylation-mediated conformational change and the loop becomes less disordered.

### Pin1 shows phosphorylation dependent interaction with pS87

**Figure 3:**
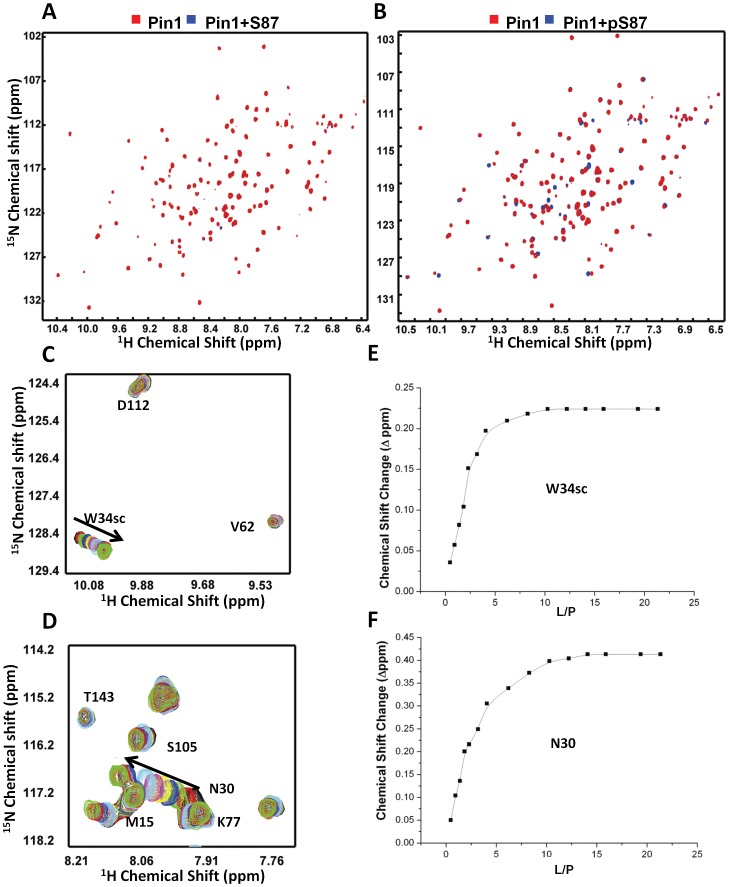
Pin 1 binds to the phosphopeptide derived from the flexible loop of Bcl-2. The ^15^N-labeled Pin1 (0.1 mM) was recorded with (A) 0.8 mM S87 and (B) phosphorylated S87 (pS87). The ^1^H-^15^N-HSQC spectra with or without peptide are show in blue and red, respectively. (C, D) Some of the amino acid chemical shifts change with the increasing peptide concentration. The arrows show the direction of chemical shift perturbations with an increasing concentration of ligand. (E, F) The relationship between chemical shift change and the ratio between ligand and protein was drawn. Values of *K_D_* were calculated from the graphs using the equation described in “Materials and methods”.

**Figure 4:**
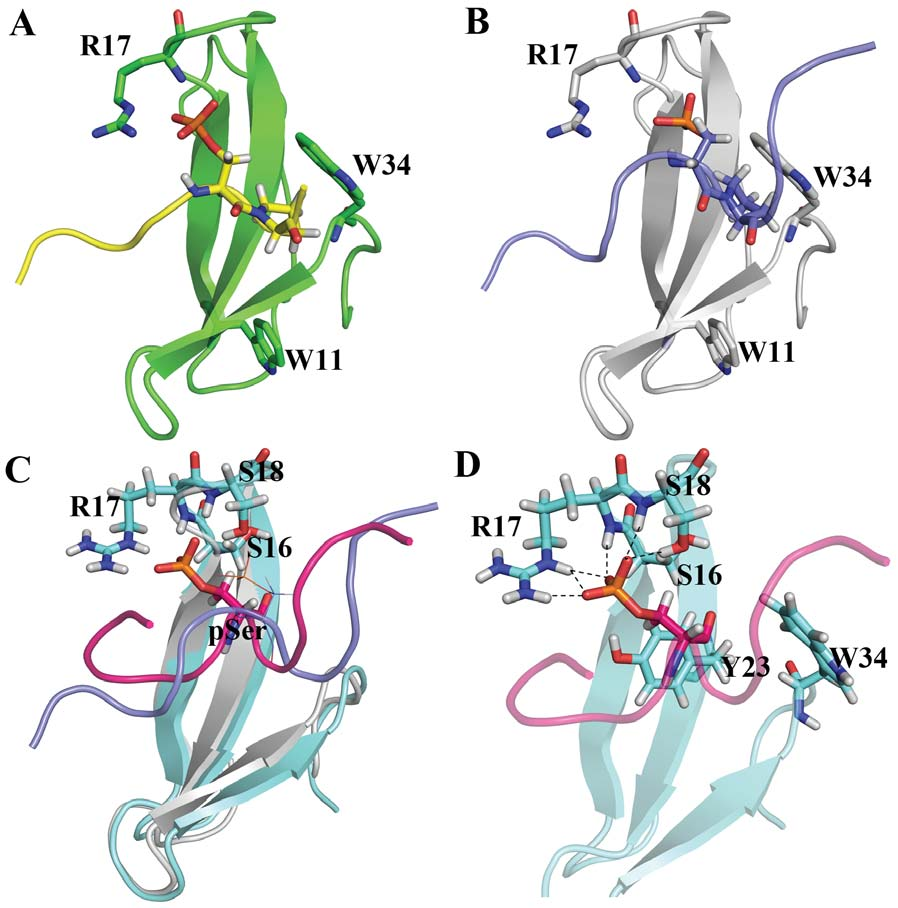
Model building and MD simulation result of Pin1-pS87 peptide. (A) Crystal structure of the Pin1 WW domain with C-terminal domain (CTD) of RNA polymerase II phosphopeptide. (B) Initial model of Pin1 WW domain with pS87 Bcl-2 phosphopeptide. Both the tryptophan residues and the phosphoserine-interacting arginine residue are highlighted as sticks, and labeled accordingly. Both phosphopeptides are highlighted with yellow (CTD peptide) and blue (pS87 Bcl-2 peptide), and the pSer-Pro motif in both the phosphopeptides is represented as sticks. (C) Initial and representative frames of MD simulation. Protein is represented with gray and cyan; pS87 peptide is highlighted with purple and pink for the initial and final frames, respectively. (D) Hydrogen bond interaction patterns of the phosphoserine with the WW domain residues. All three residues (2 serines and 1 arginine) are highlighted with sticks and labeled. Hydrogen bonds between the phosphoserine and the WW domain residues are represented with dashed lines.

It has been demonstrated that Pin1 interacts with Bcl-2, and the interaction is phosphorylation dependent [18]. Binding between the S87 peptide and Pin1 was examined by monitoring chemical shift perturbations on a 2D ^1^H-^15^N HSQC spectra of Pin1 upon addition of the peptides. The normal peptides derived from the loop region showed no interaction with pin1 (Fig. 3A,), while the phosphorylated peptides clearly showed binding to Pin1 (Fig. 3 B–D). As seen in Fig. 3, the affected residues in Pin1 were highlighted in the spectra with an increasing concentration of the pS87 peptides. Our results are consistent with previously studies showing that the WW domain of Pin1 is involved in the molecular interaction with phosphorylated peptides (Fig. S6) [32]. The calculated *K*_D_ for the binding between Pin1 and the phosphorylated Bcl-2 peptide (pS87) was about 140 µM (Fig. 3). The other peptides also showed similar phosphorylation dependent-interactions with Pin1 (Fig. S2). Analyses of the available 3D structural information (1F8A, 1I8G and 1I8H) revealed that the pSer/pThr-Pro motif forms several crucial interactions with the Pin1 WW domain. The crystal structure of the C-terminal domain (CTD) of RNA polymerase II with WW domain (PDB ID: 1F8A) disclosed the pattern of charge-charge interaction between phosphoserine and residue R17, as well as the hydrophobic cluster formed by Y23 and W34 for proline residue of pSer-Pro motif (Fig. 4A). Previous mutational studies of R17A and W34A cause 6- and 18-fold decreases in binding affinity with the CTD of RNA polymerase II peptide, respectively, suggesting that these two residues are crucial in the pSer-Pro motif of phosphopeptides. Our NMR titration studies with phosphorylated peptide are consistent with these observations.

**Figure 5:**
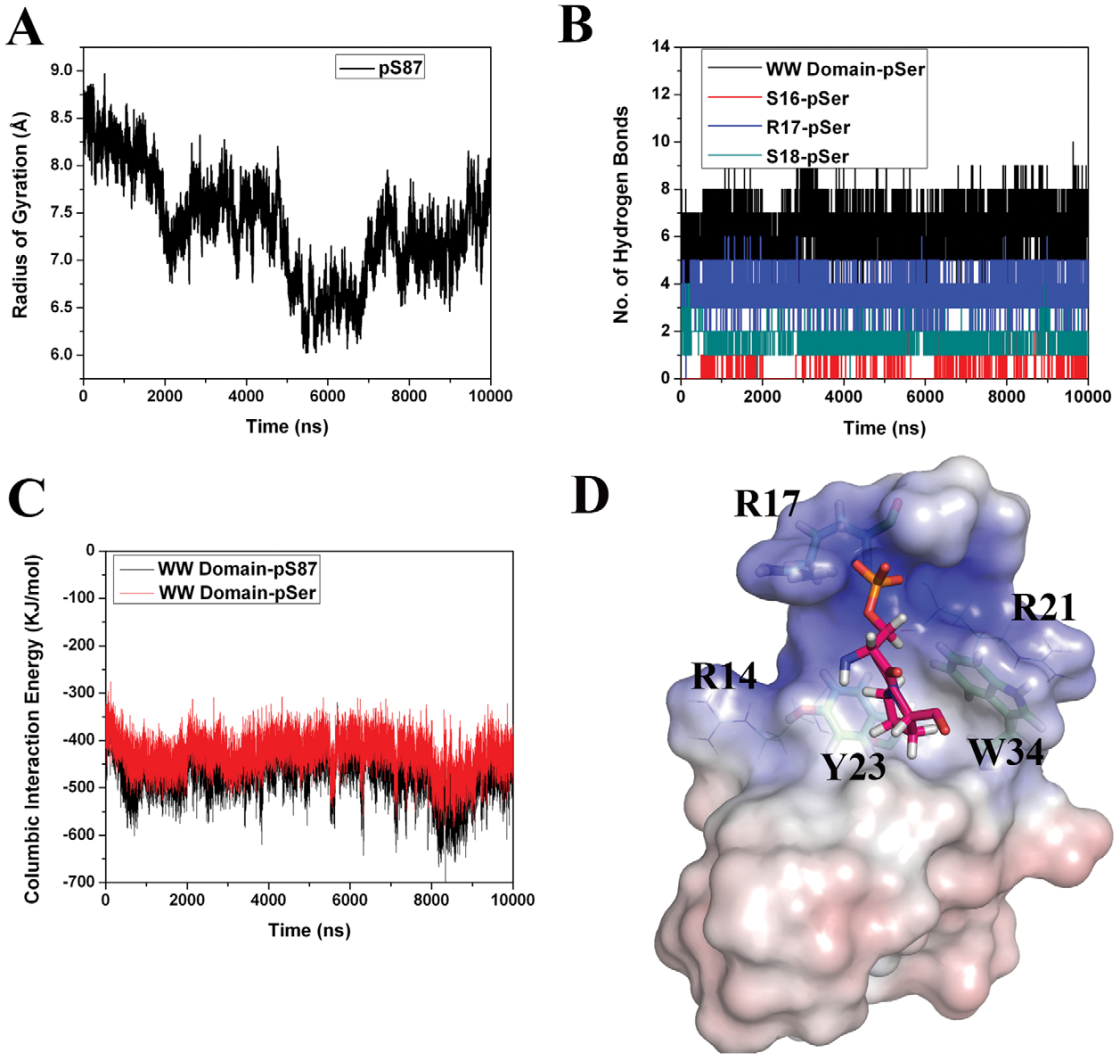
Pin1 WW domain-pS87 MD simulation analyses. (A) Radius of gyration for pS87 Bcl-2 phosphopeptide in the WW domain-pS87 simulation. (B) Hydrogen bond interaction analysis between pS87 and the WW domain residues. (C) Short range columbic interaction analysis between the WW domain and the pS87, as well as the WW domain and the pSer alone. (D) Electrostatic potential calculations of the WW domain and the pS87 phosphopeptide complex. The arginine residue, which forms a charged/hydrogen bond interaction with pSer, is highlighted as sticks, and the other two arginine residues are represented with lines. The hydrophobic pocket residues (Y23 and W34) are also shown in stick representation. The pSer-Pro motif is also highlighted.

Based on this available information, an initial complex model of the Pin1 WW domain with the Bcl-2 pS87 peptide (Fig. 4B) was generated and utilized for MD simulation studies. To understand the conformation flexibility of pS87 during simulation and to analyze the population of peptide conformers as well as obtain the representative conformer from the most populated cluster, we have carried out the cluster analyses of MD simulation snapshots. The g_cluster analysis tool of GROMACS distribution was used to generate ten clusters by single linkage method, where 77% of the frames were populated in a single cluster (Fig. S3). The representative/average conformer obtained from this large cluster was considered for further analyses. Comparison of the initial and the representative frames revealed that the phosphorylated peptide undergoes a conformational change, as observed in the CD analyses, for the peptides solved by NMR, and for the peptide-alone simulations (Fig. 4C). The Rg analysis of pS87 peptide in the complex simulation also disclosed the same change (Fig. 5A). In total, 6–7 hydrogen bonds were observed between the pS87 peptide and the WW domain, and all the interactions were formed by the phosphoserine residue (Fig. 5D). R17 was the major contributor in binding with the peptide, forming 3–4 hydrogen bonds with the phosphoserine of the peptide (Figure 5B), and residues S16 and S18 also formed reasonably stable interactions with the phosphoryl group. The interaction energy analyses disclosed that the WW domain- pS87 complex is stabilized majorly by hydrophilic interactions as well as hydrophobic interactions. The proline residue present in the pSer-Pro motif formed stable hydrophobic interactions with two well-conserved residues, Y23 and W34. The columbic interaction energy analysis revealed that the stable interaction energy between the WW domain and the phosphopeptide was achieved from the phosphoserine residue interaction, which is consistent with previous experimental results (Fig. 5C). Three positively charged residues (R14, R17 and R21) formed a complementary surface for the negatively charged phosphoserine residue of pS87, as shown by electrostatic calculation results. The residues, Y23 and W34, form a hydrophobic patch for the proline residue in the pSer-Pro motif (Fig. 5D).

## Discussion

The anti-apoptotic protein Bcl-2 plays an important role in the regulation of apoptosis. Post-translational modification of Bcl-2 affects its function and the consequences of Bcl-2 phosphorylation on its function are still controversial [33]. The residues T56, S70, T74, and S87 in the flexible loop of Bcl-2 have been shown to be phosphorylated in response to a variety of external stimuli [12]. In this work, to better understand the molecular basis of the Bcl-2 phosphorylation, we first studied the characteristics of the Bcl-2 phosphorylation by JNK and ERK2, which are consistently shown to be involved in the phosphorylation of Bcl-2 [29], [34]. From our studies on the kinase specificity using the Bcl-2 mutants and the flexible loop-derived peptides, we suspect that JNK and ERK2 show substrate specificity with the four known phosphorylation sites. Our results indicate that S87 is the primary phosphorylation site for JNK and ERK2, with S70 and T74 being poorer substrates for both kinases.

The presence of a consensus substrate sequence is important for its recognition as a substrate by a specific protein kinase, since the specificity-determining characteristics of the phosphorylation site are contained in the flanking amino acids of the target phosphorylation site [35]. Previously, it has been shown that the substrate consensus sequence for ERK2 is PXaan(S/T)P, where Xaa is a neutral or basic amino acid and n = 1 or 2 residues [36]. The analysis of the primary sequences of the known phosphorylation sites in Bcl-2 (Table 1) revealed that the flanking sequence of the S87 appears to be conserved and consistent with the consensus sequence described above. On the other hand, our studies on Bcl-2Δ(V35–D79), which contains only the S87 phosphorylation site, showed less-efficient phosphorylation, as compared with the pS87 peptide (Fig. S1B, C). We speculate that this could be attributed to the lack of a region in the loop domain of Bcl-2, which might be able to interact with the common docking site of ERK2 and contribute to a better substrate specificity for ERK2, as exemplified in the case of the bipartite recognition model of ERK2 and its substrate MKP3 [37].

Proteins containing regions of denatured or random coil structures do not exhibit a long half-life due to cleavage by cellular proteases [38]. The loop of Bcl-2 might be recognized and protected by certain proteins, and subsequently the molecular interactions would stabilize Bcl-2 and result in maintenance of the anti-apoptotic function of Bcl-2. Based on the CD data, NMR experiments and MD simulations in this study, we demonstrated that Bcl-2 undergoes a conformational change upon phosphorylation (Fig. 1 and 2) to allow Bcl-2 to be competent for binding to Pin1.

## Supporting Information

Figure S1 **Phosphorylation of Bcl-2 in the loop domain by ERK2 and JNK.** (A–B) Different peptides (200 µM) were added into the reaction mixture with either (A) JNK (10 µM) or (B) ERK2 (10 µM). After reaction at 30°C for 1 h, the samples were heated at 90°C to inactivate the kinase and loaded onto C-18 column for reverse phase-HPLC analysis. (C) Point mutations of Bcl-2 (T56A and S87A) were used for the kinase reaction as described in “Materials and methods”, and the phosphorylation reactions were analyzed by using γ-^32^P-ATP and followed by autoradiography. (D) Western blotting shows the expression levels of Bcl-2 and its point mutants used in the kinase reactions. (E) The wild-type Bcl-2 and the flexible loop-deletion Bcl-2 mutant Bcl-2, Δ(V35–V89):6A were subject to the phosphorylation reactions by ERK2 and JNK. 1, Bcl-2 with ERK2; 2, Bcl-2Δ(V35–V89):6A with ERK2; 3, Bcl-2 with JNK; 4, Bcl-2Δ(V35–V89):6A with JNK.
(TIF)

Figure S2 **Structures of the peptides from Bcl-2.** Ensembles of 10 lowest energy NMR structures for T74 (A), T56 (B), p74 (C) and pT56 peptides (D) are shown, respectively. Residues Thr is highlighted with sticks. The ^15^N-labeled Pin1 was recorded in the presence of regular/phosphorylated T74 (E) and T56 (F). 2D ^1^H-^15^N HSQC spectra with or without peptide are shown in red and black, respectively.
(TIF)

Figure S3 **Clustering analyses of MD simulation data based on RMSD of the pS87 Bcl-2 phosphopeptide.** Highest number of conformations is populated in Cluster-4.
(TIF)

Figure S4 **Model for the interaction between Pin1 and pS87** (A) S87 peptide. (B) The pS87 peptide, which contains the pSer-Pro motif, undergoes conformational changes after phosphorylation. (C) Structural model of the Pin 1 WW domain complexed with pS87 was generated using GOLD v3.1.1 (Cambridge Crystallographic Data Centre, UK). (D) The structure of the Pin1 WW domain complexed with Tau peptide (PDB 1I8G). The pSer-Pro motif is shown in red. The model shows that phosphorylation of the Bcl-2 S87 peptide undergoes a conformational change and the resulting structure resembles the complex structure of the WW domain and the Tau peptide.
(TIF)

Figure S5 **CSI analysis of S87 and pS87.** Chemical shift index analysis for the peptides. The H^α^ chemical shifts were compared with that of the random coil of each residue.
(TIF)

Figure S6 **The amide region of the peptides.** 1D proton NMR was collected as described in the “Materials and methods”. The superimposed spectra in the amide proton region are shown. Peptide and phosphorylated peptide are shown as blue and red, respectively. The residue numbers are shown on the spectra.
(TIF)

Table S1 Resonance assignment of peptides S87 and pS87.
(DOC)
