# Supplementary figures and images for: The Natively Disordered Loop of Bcl-2 Undergoes Phosphorylation-Dependent Conformational Change and Interacts with Pin1
Source: PLoS One. 2012 Dec 18;7(12):e52047. doi: 10.1371/journal.pone.0052047 (PMC3525568; doi:10.1371/journal.pone.0052047)

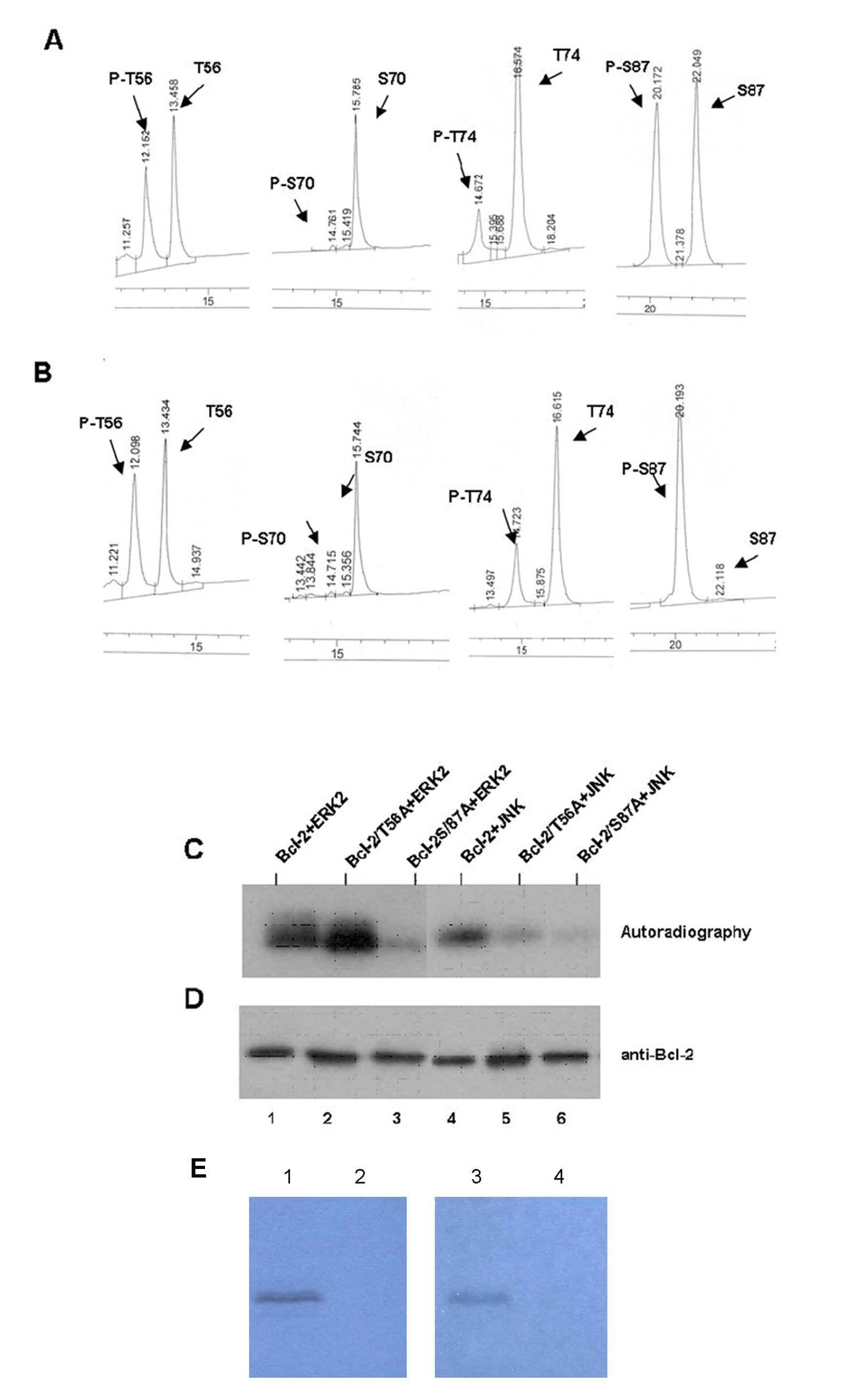

Supplement: Figure S1 — Phosphorylation of Bcl-2 in the loop domain by ERK2 and JNK. (A–B) Different peptides (200 µM) were added into the reaction mixture with either (A) JNK (10 µM) or (B) ERK2 (10 µM). After reaction at 30°C for 1 h, the samples were heated at 90°C to inactivate the kinase and loaded onto C-18 column for reverse phase-HPLC analysis. (C) Point mutations of Bcl-2 (T56A and S87A) were used for the kinase reaction as described in “Materials and methods”, and the phosphorylation reactions were analyzed by using γ-32P-ATP and followed by autoradiography. (D) Western blotting shows the expression levels of Bcl-2 and its point mutants used in the kinase reactions. (E) The wild-type Bcl-2 and the flexible loop-deletion Bcl-2 mutant Bcl-2, Δ(V35–V89):6A were subject to the phosphorylation reactions by ERK2 and JNK. 1, Bcl-2 with ERK2; 2, Bcl-2Δ(V35–V89):6A with ERK2; 3, Bcl-2 with JNK; 4, Bcl-2Δ(V35–V89):6A with JNK. (TIF) [file pone.0052047.s001.tif]

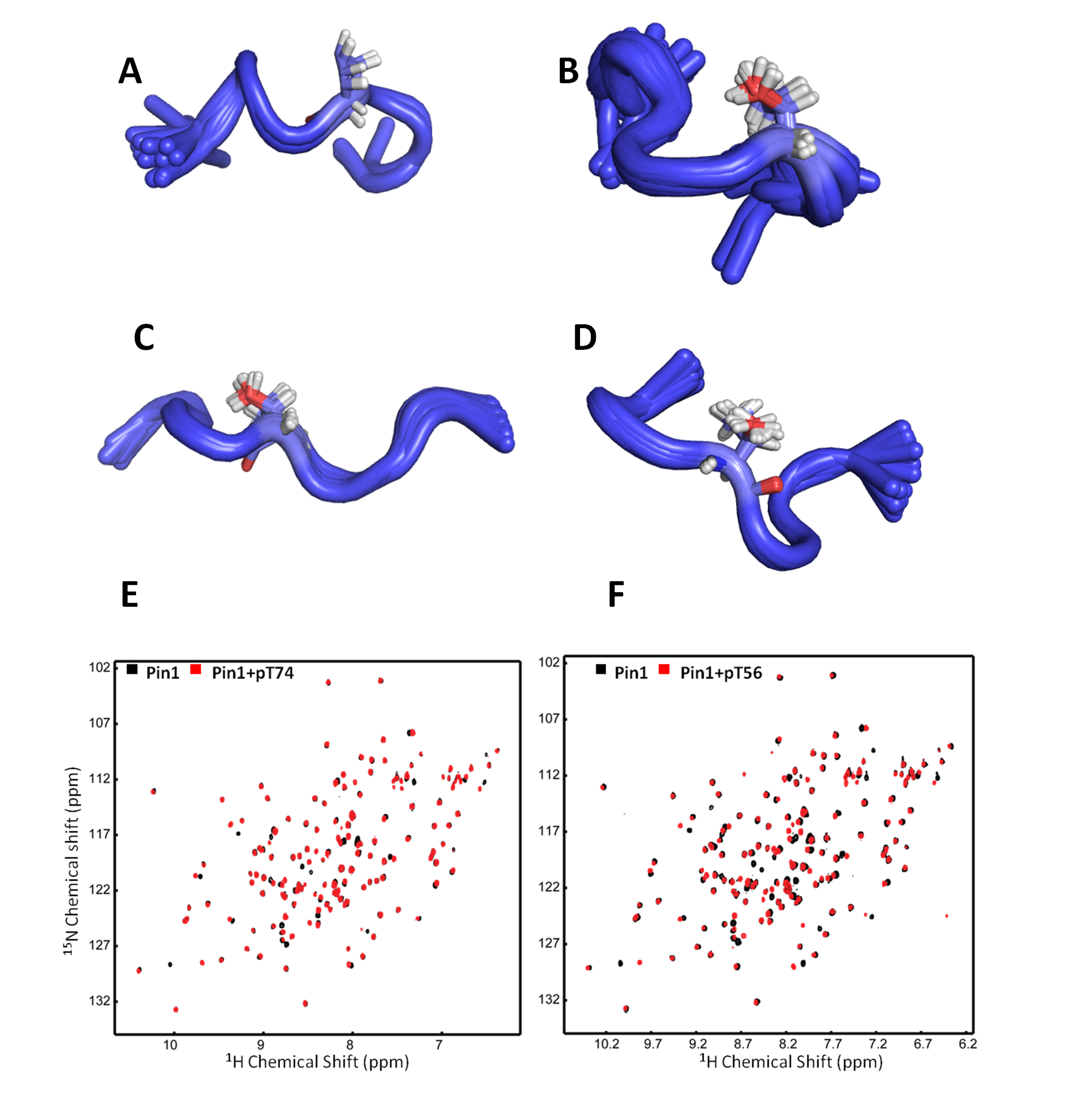

Supplement: Figure S2 — Structures of the peptides from Bcl-2. Ensembles of 10 lowest energy NMR structures for T74 (A), T56 (B), p74 (C) and pT56 peptides (D) are shown, respectively. Residues Thr is highlighted with sticks. The 15N-labeled Pin1 was recorded in the presence of regular/phosphorylated T74 (E) and T56 (F). 2D 1H-15N HSQC spectra with or without peptide are shown in red and black, respectively. (TIF) [file pone.0052047.s002.tif]

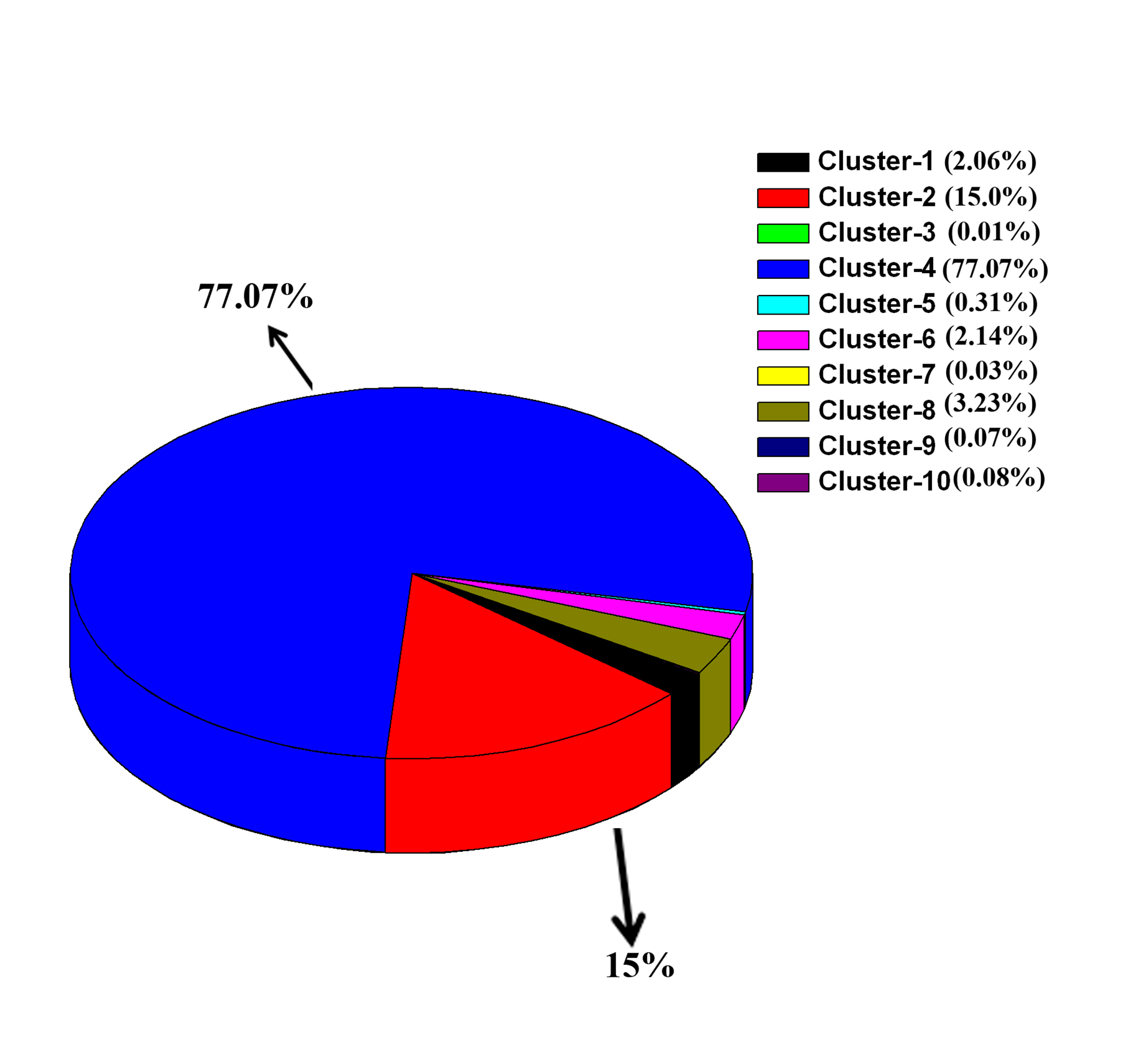

Supplement: Figure S3 — Clustering analyses of MD simulation data based on RMSD of the pS87 Bcl-2 phosphopeptide. Highest number of conformations is populated in Cluster-4. (TIF) [file pone.0052047.s003.tif]

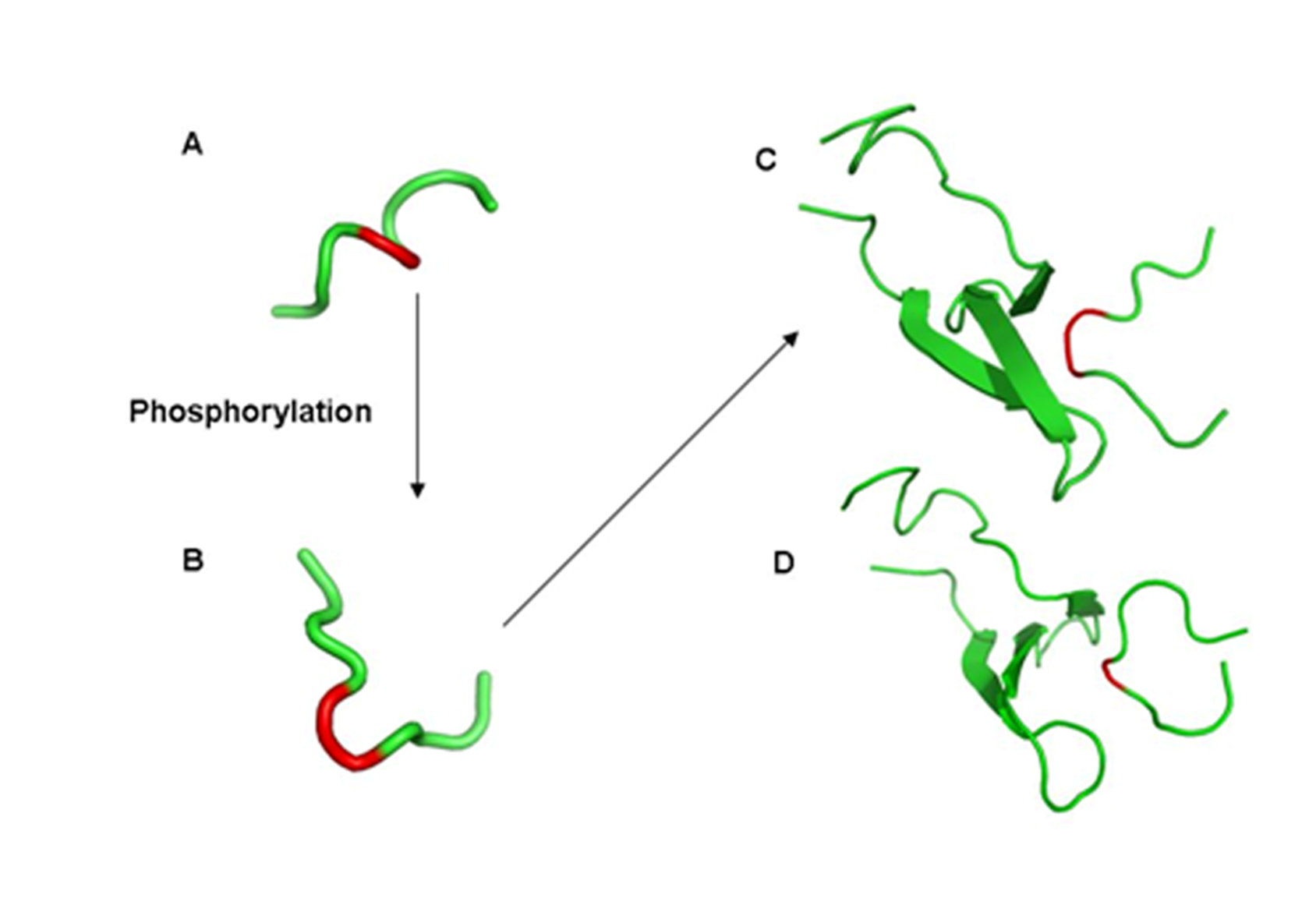

Supplement: Figure S4 — Model for the interaction between Pin1 and pS87 (A) S87 peptide. (B) The pS87 peptide, which contains the pSer-Pro motif, undergoes conformational changes after phosphorylation. (C) Structural model of the Pin 1 WW domain complexed with pS87 was generated using GOLD v3.1.1 (Cambridge Crystallographic Data Centre, UK). (D) The structure of the Pin1 WW domain complexed with Tau peptide (PDB 1I8G). The pSer-Pro motif is shown in red. The model shows that phosphorylation of the Bcl-2 S87 peptide undergoes a conformational change and the resulting structure resembles the complex structure of the WW domain and the Tau peptide. (TIF) [file pone.0052047.s004.tif]

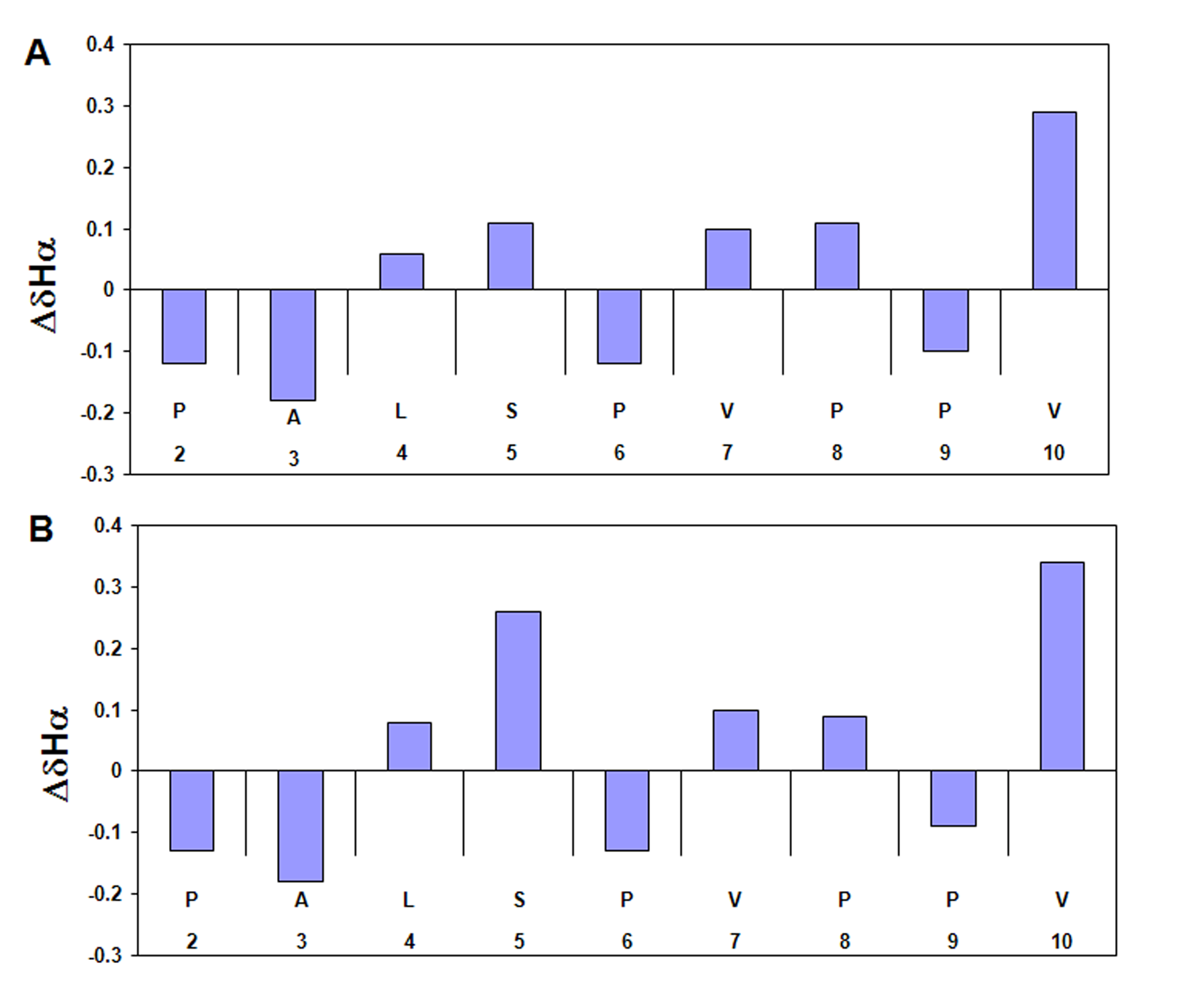

Supplement: Figure S5 — CSI analysis of S87 and pS87. Chemical shift index analysis for the peptides. The Hα chemical shifts were compared with that of the random coil of each residue. (TIF) [file pone.0052047.s005.tif]

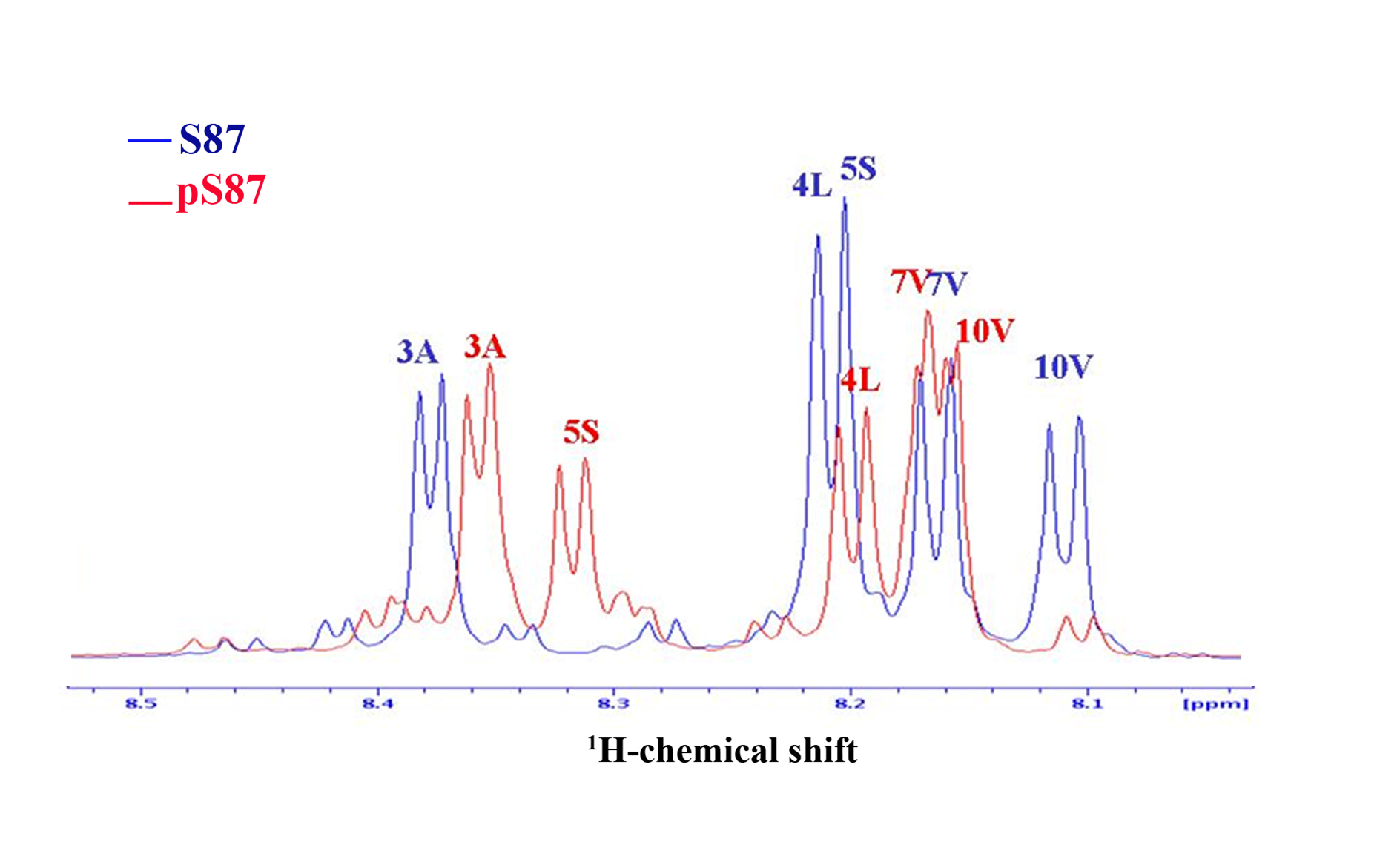

Supplement: Figure S6 — The amide region of the peptides. 1D proton NMR was collected as described in the “Materials and methods”. The superimposed spectra in the amide proton region are shown. Peptide and phosphorylated peptide are shown as blue and red, respectively. The residue numbers are shown on the spectra. (TIF) [file pone.0052047.s006.tif]
