# Supplementary material for: The Natively Disordered Loop of Bcl-2 Undergoes Phosphorylation-Dependent Conformational Change and Interacts with Pin1
Source: PLoS One. 2012 Dec 18;7(12):e52047. doi: 10.1371/journal.pone.0052047 (PMC3525568; doi:10.1371/journal.pone.0052047)
Supplement: Table S1 — Resonance assignment of peptides S87 and pS87. (DOC) [file pone.0052047.s007.doc]

**Table S1** Resonance assignment of peptides S87 and pS87

| Residues | S87 | |  | pS87 | |
| --- | --- | --- | --- | --- | --- |
| Pro | 2.HA | 4.318 |  | 2.HA | 4.312 |
|  | 2.HB1 | 2.162 |  | 2.HB1 | 2.157 |
|  | 2.HG2 | 1.807 |  | 2.HG2 | 1.784 |
|  | 2.HG1 | 1.858 |  | 2.HG1 | 1.865 |
|  | 2.HD2 | 3.424 |  | 2.HD2 | 3.424 |
|  | 2.HD1 | 3.425 |  | 2.HD1 | 3.422 |
| Ala | 3.HN | 8.381 |  | 3.HN | 8.364 |
|  | 3.HA | 4.165 |  | 3.HA | 4.171 |
|  | 3.HB1 | 1.241 |  | 3.HB1 | 1.239 |
| Leu | 4.HN | 8.213 |  | 4.HN | 8.179 |
|  | 4.HA | 4.234 |  | 4.HA | 4.246 |
|  | 4.HB1 | 1.412 |  | 4.HB1 | 1.488 |
|  | 4.HG | 1.476 |  | 4.HG | 1.478 |
|  | 4.HD11 | 0.752 |  | 4.HD11 | 0.81 |
| Ser | 5.HN | 8.211 |  | 5.HN | 8.322 |
|  | 5.HA | 6.406 |  | 5.HA | 4.762 |
|  | 5.HB2 | 3.658 |  | 5.HB2 | 3.908 |
|  | 5.HB1 | 3.73 |  | 5.HB1 | 3.992 |
| Pro | 6.HA | 4.324 |  | 6.HA | 4.308 |
|  | 6.HB2 | 1.929 |  | 6.HB2 | 2.115 |
|  | 6.HB1 | 2.121 |  | 6.HG2 | 1.756 |
|  | 6.HG2 | 1.882 |  | 6.HG1 | 1.88 |
|  | 6.HG1 | 1.75 |  | 6.HD2 | 3.593 |
|  | 6.HD2 | 3.675 |  | 6.HD1 | 3.661 |
|  | 6.HD1 | 3.584 |  | 7.HN | 8.167 |
| Val | 7.HN | 8.169 |  | 7.HA | 4.052 |
|  | 7.HA | 4.053 |  | 7.HB | 2.022 |
|  | 7.HB | 2.027 |  | 7.HG11 | 0.82 |
|  | 7.HG11 | 0.829 |  | 8.HA | 4.535 |
| Pro | 8.HA | 4.551 |  | 8.HB2 | 1.914 |
|  | 8.HB2 | 1.929 |  | 8.HB1 | 2.218 |
|  | 8.HB1 | 2.22 |  | 8.HG1 | 1.889 |
|  | 8.HG2 | 1.859 |  | 8.HD2 | 2.633 |
|  | 8.HG1 | 1.738 |  | 8.HD1 | 3.783 |
|  | 8.HD2 | 3.546 |  | 9.HA | 4.353 |
|  | 8.HD1 | 3.774 |  | 9.HB2 | 1.907 |
| Pro | 9.HA | 4.345 |  | 9.HB1 | 2.14 |
|  | 9.HB1 | 2.145 |  | 9.HG2 | 1.866 |
|  | 9.HG2 | 1.882 |  | 9.HG1 | 1.784 |
|  | 9.HG1 | 1.789 |  | 9.HD2 | 3.517 |
|  | 9.HD2 | 3.539 |  | 9.HD1 | 3.684 |
|  | 9.HD1 | 3.679 |  | 10.HN | 8.17 |
| Val | 10.HN | 8.116 |  | 10.HA | 4.29 |
|  | 10.HA | 4.245 |  | 10.HB | 1.916 |
|  | 10.HB | 1.914 |  | 10.HG11 | 0.82 |

The resonance assignment was assigned based upon the TOCSY and ROESY experiments at 298 K. The atom names and chemical shifts are shown.
